# Potent α-glucosidase and α-amylase inhibitory activities of standardized 50% ethanolic extracts and sinensetin from *Orthosiphon stamineus* Benth as anti-diabetic mechanism

**DOI:** 10.1186/1472-6882-12-176

**Published:** 2012-10-08

**Authors:** Elsnoussi Ali Hussin Mohamed, Mohammad Jamshed Ahmad Siddiqui, Lee Fung Ang, Amirin Sadikun, Sue Hay Chan, Soo Choon Tan, Mohd Zaini Asmawi, Mun Fei Yam

**Affiliations:** 1 School of Pharmaceutical Sciences, Universiti Sains Malaysia, Pulau Pinang 11800, Malaysia; 2 Kulliayya of Pharmacy, International Islamic University Malaysia, Kuantan, Pahang 25200, Malaysia

## Abstract

**Background:**

In the present study, we tested a 50% ethanolic extract of *Orthosiphon stamineus* plants and its isolated bioactive compound with respect to their α-glucosidase and α-amylase inhibitory activities.

**Methods:**

Bioactive flavonoid sinensetin was isolated from 50% ethanolic extract of *Orthosiphon stamineus*. The structure of this pure compound was determined on the NMR data and the α-glucosidase and α-amylase inhibitory activities of isolated sinensetin and 50% ethanolic extract of *Orthosiphon stamineus* were evaluated.

**Results:**

*In vitro* studies of a 50% ethanolic extract of *O. stamineus* and the isolated sinensetin compound showed inhibitory activity on α-glucosidase (IC_50_: 4.63 and 0.66 mg/ml, respectively) and α-amylase (IC_50_: 36.70 mg/ml and 1.13 mg/ml, respectively). Inhibition of these enzymes provides a strong biochemical basis for the management of type 2 diabetes via the control of glucose absorption.

**Conclusion:**

Alpha-glucosidase and α-amylase inhibition could the mechanisms through which the 50% ethanolic extract of *O. stamineus* and sinensetin exert their antidiabetic activity, indicating that it could have potential use in the management of non-insulin-dependent diabetes.

## Background

The number of diabetic patients is rapidly rising in most parts of the world, especially in developing countries such as Thailand, India and Indonesia. In general, the control of blood glucose concentrations near the normal range is mainly based on the use of oral hypoglycaemic/antihyperglycaemic agents and insulin. However, all of these treatments have limited efficacy and are associated with undesirable side effects [1-3], leading to increasing interest in the use of medicinal plants for the alternative management of type 2 diabetes mellitus. The control of postprandial plasma glucose levels is critical in the early treatment of diabetes mellitus and for reducing chronic vascular complications [4]. A sudden increase in blood glucose levels, which causes hyperglycaemia in type 2 diabetes patients, occurs due to the hydrolysis of starch by pancreatic α-amylase and the uptake of glucose by intestinal α-glucosidases [5]. An effective strategy for type 2 diabetes management is the strong inhibition of intestinal α-glucosidases and the mild inhibition of pancreatic α-amylase [6]. One therapeutic approach for reducing postprandial hyperglycaemia in patients with diabetes mellitus is preventing the absorption of carbohydrates after food uptake. Only monosaccharides, such as glucose and fructose, can be transported out of the intestinal lumen into the bloodstream. Complex starches, oligosaccharides and disaccharides must be broken down into individual monosaccharides before they are absorbed in the duodenum and upper jejunum. This digestion is facilitated by enteric enzymes, including pancreatic α-amylase and α-glucosidases attached to the brush border of intestinal cells.

Herbal medicines have received a great deal of attention as alternative medicines in recent years in Malaysia and are sold as dietary supplements. One of the local herbs, scientifically known as *Orthosiphon stamineus* Benth (Lamiaceae) or locally called misai kucing, has attracted much attention for research purposes. *Orthosiphon stamineus* is also found in other countries such as Thailand, Indonesia and Europe. In these countries, misai kucing is also known as yaa nuat maeo, rau meo or cay bac (Thailand), kumis kucing or remujung (Indonesia), moustaches de chat (French) and Java tea (European) [7]. It is widely used for the treatment of many diseases, especially those affecting the urinary tract, diabetes mellitus, hypertension, rheumatism, tonsillitis and menstrual disorders. The methanolic extracts of this plant have shown inhibitory activity on nitric oxide production in macrophage-like cells [8,9]. Several classes of compounds have been identified in this plant, including flavonoids, terpenoids, saponins, hexoses, organic acids, caffeic acid derivatives, chromene and myo-inositol [10-14]. The most important components of *O. stamineus* leaves are the polyphenols, the polymethoxylated flavonoids: sinensetin and eupatorine, etc., and the caffeic acid derivatives: rosmarinic acid, cichoric acid and caffeic acid, etc. [14]. Intestinal glycosidase enzymes play an important role in carbohydrate digestion and absorption. Therefore, an inhibitor of intestinal glucosidase could be expected to retard carbohydrate digestion and absorption. One reasonable method of controlling these carbohydrate–dependent diseases would be to limit intestinal carbohydrate digestion. It has been recognized that α-glucosidase inhibitors can be used to prevent certain disorders such as diabetes, obesity, hyperlipidaemia and hyperlipoproteinaemia.

We previously reported that the *O. stamineus* was able to reduced blood glucose in STZ-induced diabetic rat. In the same study we reported sinensetin, eupatorin and 3′-hydroxy-5,6,7,4′-tetramethoxyflavone are present in *O. stamineus*[15-17]. Although many *O. stamineus* have been per-formed, a clear antidiabetic mechanism of action for this plant has not yet been established. To the best of our knowledge there have been no previous reports on the *in vitro* inhibitory activity of the *O. stamineus* plant on α-glucosidase or α-amylase and this inhibitory effect could be one of the mechanisms of action of anti-diabetic effect of *O. stamineus.* The aim of the present study was to evaluate the *in vitro* effects of a 50% ethanolic extract of *O. stamineus* and its identified compound sinensetin on α-glucosidase and α-amylase enzyme inhibition compared to their previously reported antihyperglycaemic effects in normal and STZ-induced diabetic rats [15] in order to contribute to the understanding of their mechanisms of action.

## Methods

### Plant materials and preparation of the samples

Leaves of *Orthosiphon stamineus* were obtained from Kepala Batas, Pulau Pinang Malaysia. The plant was identified by the School of Biological Sciences, Universiti Sains Malaysia, and a voucher specimen (10810) was deposited at the Herbarium of the School of Biological Sciences, Universiti Sains Malaysia. The dried leaves were powdered using a milling machine and were extracted with 50% (v/v) ethanol using the maceration method (200 g dried leaves in 2 l of 50% ethanol at 55°C for 24 h; two cycles) for 7 days. The extracts were filtered and concentrated at 60°C using a rotary evaporator (Buchi Labortechnik, Flawil, Switzerland). Finally, the concentrated extract was freeze-dried (Labconco Corporation, Kansas City, MO, USA) to yield 10.3% dry powder.

### HPLC study of the 50% ethanolic extract of *O. stamineus*

High-performance liquid chromatography (HPLC) analysis was performed using a Shimadzu-LC system (Shimadzu, Japan) equipped with a CBM-20A controller, LC-20AT pump, DGU-20A5 degasser, SIL-20A auto-sampler, SPD-20AV detector and a CTO-10ASvp column oven.

Chromatographic separations were achieved using an Agilent Eclipse Plus C18 (250 × 4.6 mm i.d.; 5 μm). A Zorbax guard fittings kit packed with a replaceable Eclipse Plus C18 Guard column (12.5 × 4.6 mm i.d.; 5 μm) was used to protect the analytical column. A reverse-phase HPLC assay was carried out using an isocratic system at a flow rate of 1 ml/min, a column temperature of 25°C and a mobile phase of acetonitrile, isopropyl alcohol and 0.02 M phosphate buffer (NaH_2_PO_4_) (30:15:55 v/v) with the pH adjusted to pH 3.5 using 85% phosphoric acid. The UV detection was set at 340 nm. The injection volume was 20 μl of solution. The total run time was less than 20 min for each injection. Data were acquired and processed using LC-Solution Software. The peaks were detected at 340 nm and identified using standard substances, namely, sinensetin, eupatorin and 3′-hydroxy-5,6,7,4′-tetramethoxyflavone. The 50% ethanol extract of *O. stamineus* at 10 mg/ml was used as a stock solution. The stock solution was then diluted with the mobile phase to 1 mg/ml as the sample solution. The total amounts of 3′hydroxy-5,6,7,4′-tetramethoxyflavone, sinensetin and eupatorin (purchase from Indofine Chemical Company, NJ, USA) in the 50% ethanol extract of *O. stamineus* were quantified using developed HPLC method (n = 3). The contents of these three compounds were expressed as percentages of the dried extract [18].

### Isolation of the compounds

The 50% ethanolic extract of *O. Stamineus* was separated into ethyl acetate, butanol and water-soluble fractions. The ethyl acetate fraction with antihyperglycaemic activity was separated via silica-gel column chromatography to give two sub-fractions: namely ESF-1 (non-active) and ESF-2 (active) (ethyl acetate fraction and sub-fraction ESF-2 were active in antihyperglycaemic effect, unpublished result). The ESF-2 was fraction was then subjected to silica gel chromatography for bioactive compound isolation [19]. Isolated compound was subjected NMR study [19]. All the spectroscopic data in good agreement with Yam *et al*, 2010, it can be concluded that the isolated compound was sinensetin [19].

### *In vitro* α-glucosidase inhibition study

The α-glucosidase was assayed using a method modified by Apostolidis *et al*[20], where a 50% ethanolic extract of *O. stameinus* (62.5, 31.25, 15.6, 7.8, 3.9, and 1.95 mg/ml in dimethylsulphoxide:distilled water (1:1) solution) and sinensetin (2.5, 1.25, 0.625, and 0.3125 mg/ml in methanol) were prepared. Then, 50 μl of 50% ethanolic extract of *O. stameinus* or sinensetin solution will mixed well with 100 μl of 0.1 M phosphate buffer (pH 6.9) containing α-glucosidase (Sigma, St. Louis, USA) solution (1.0 U/ml) and the mixture were then incubated in 96-well plates at 25°C for 10 min. After pre-incubation, 50 μl of 5 mM p-nitrophenyl-α-D-glucopyranoside (Sigma, St. Louis, USA) solution in 0.1 M phosphate buffer (pH 6.9) was added to each well at timed intervals. The reaction mixtures were incubated at 25°C for 5 min. Before and after incubation absorbance readings were recorded at 405 nm using a micro-plate reader (Thermomax, Molecular device Co., Virginia, USA) and compared to a control which contained 50 μl of the buffer solution instead of the extract. The experiments were performed in triplicate and the α-glucosidase inhibitory activity was expressed as percentage inhibition. Acarbose (Bayer Pharmaceuticals, Leverkusen, Germany) was prepared in distilled water at 10, 5, 2.5 and 1.25 mg/ml were made and used as positive controls.

### Alpha-amylase inhibition assay

The inhibition of α-amylase was determined using an assay modified from the Worthington Enzyme Manual [21], where a 50% ethanolic extract of *O. stameinus* (62.5, 31.25, 15.6, 7.8, 3.9, and 1.95 mg/ml in dimethylsulphoxide:distilled water (1:1) solution) and sinensetin (2.5, 1.25, 0.625, and 0.3125 mg/ml in methanol) were prepared. A total of 500 μl of each concentration of extract and sinensetin mixed with and 500 μl of 0.02 M sodium phosphate buffer (pH 6.9) containing α-amylase (Sigma, St. Louis, USA) solution (0.5 mg/ml) and incubated at 25°C for 10 min. After pre-incubation, 500 μl of a 1% starch solution in 0.02 M sodium phosphate buffer (pH 6.9) was added to each tube at timed intervals. The reaction mixtures were then incubated at 25°C for 10 min. The reaction was stopped with 1.0 ml of dinitrosalicylic acid (Sigma, St. Louis, USA) colour reagent. The test tubes were then incubated in a boiling water bath for 5 min and cooled to room temperature. The reaction mixture was then diluted after adding 15 ml of distilled water, and the absorbance was measured at 540 nm using a micro-plate reader (Thermomax, Molecular device Co., Virginia, USA). The experiments were performed in duplicate and the absorbance of sample blanks (buffer instead of enzyme solution) and a control (buffer in place of sample extract) were also recorded. The absorbance of the final extract was obtained by subtracting its corresponding sample blank reading. Sinensitin was dissolved in methanol and serial dilutions of 2.5, 1.25, 0.62 and 0.31 mg/ml were prepared. Acarbose was prepared in distilled water at 10, 5, 2.5 and 1.25 mg/ml were made and used as positive controls.

### Statistical analysis

All the data were showed as mean ± SD and statistical analysis were performed using one-way analysis of variance (ANOVA) followed by Tukey multiple comparison test with *P* < 0.05 taken as significant (GraphPad Prism 5.0).

## Results

### Phytochemical composition

**Figure 1:**
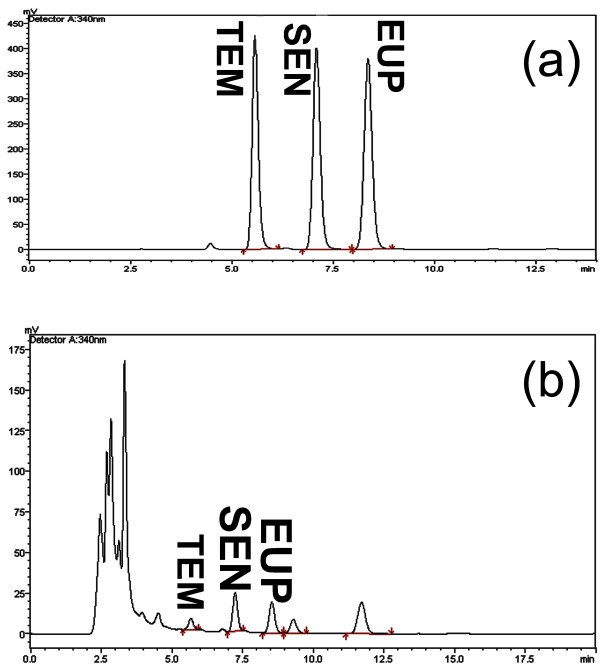
**HPLC chromatogram of standard markers. Peaks: (a) Standard TMF (3′-hydroxy-5,6,7,4′-tetramethoxyflavone), SEN (sinensetin) and EUP (eupatorin). **(**b**): HPLC chromatogram of 50% ethanolic extract of *O. stamineus*.

The HPLC results showed that the percentages of 3′hydroxy-5,6,7,4′-tetramethoxyflavone, sinensetin and eupatorin in the 50% ethanolic extract of *O. Stamineus* were 1.0150 ± 0.0007%, 3.7642 ± 0.0188%, and 3.0315 ± 0.0252%, respectively (Figure 1).

### *In vitro* α-glucosidase inhibition study

**Figure 2:**
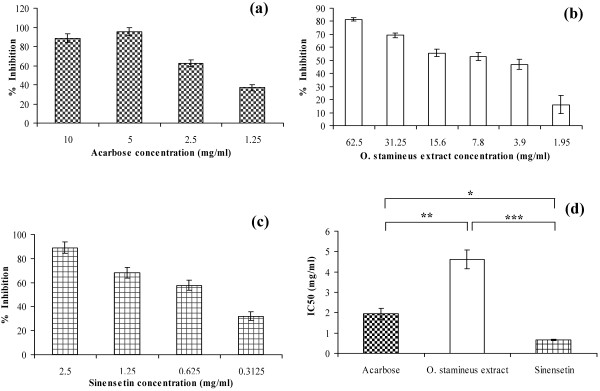
**Percentage inhibition of acarbose (a), 50% ethanol extract of *O. stamineus *(b) and sinensetin (c) and IC**_**50 **_**of treatment groups (d) on α-glucosidase enzyme *in vitro*. Result represented as mean ± S.D. of percent enzyme activity.**

The *in vitro* α-glucosidase inhibition study showed that both the 50% ethanol extract of *O. stamineus* and sinensetin inhibited α-glucosidase. The percentage inhibition at the α-glucosidase concentrations of 62.5, 31.25, 15.6, 7.8, 3.9 and 1.95 mg/ml by the 50% ethanol extract of *O. stamineus* showed a concentration-dependent reduction. The highest concentration of 62.5 mg/ml showed a maximum inhibition of nearly 81.4%, while the lowest concentration of 1.95 mg/ml showed a minimum inhibition of nearly 16.2% (Figure 2(b)). Sinensetin showed strong inhibition percentages ranging from 89.0–32.0% for α-glucosidase concentrations ranging from 2.5–0.31 mg/ml (Figure 2(c)). The 50% ethanolic extract of *O. stamineus* was less potent in inhibiting α-glucosidase compared to acarbose and sinensetin. Acarbose was found to be the most active with a half maximal inhibitory concentration (IC_50_) of 1.93 ± 0.281 mg/ml. The IC_50_ values of the 50% ethanolic extract of *O. stamineus* and sinensetin were 4.63 ± 0.413 and 0.66 ± 0.025 mg/ml, respectively (Figure 2(d)). IC_50_ values of sinensetin was significantly lower than acarbose and 50% ethanolic extract of *O. stamineus* at *P* < 0.01.

### *In vitro* α-amylase inhibition study

**Figure 3:**
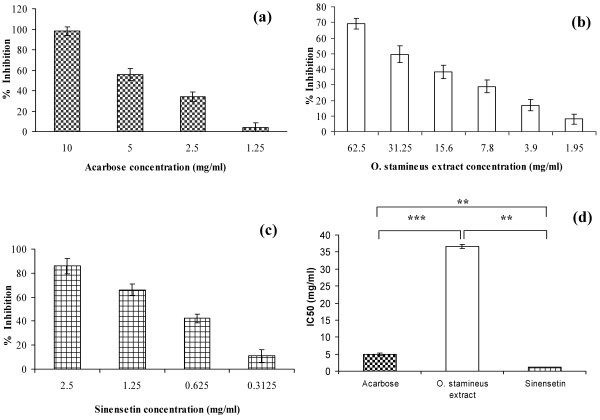
**Percentage inhibition of acarbose (a), 50% ethanol extract of *O. stamineus *(b) and sinensetin (c) and IC**_**50 **_**of treatment groups (d) on α-amylase enzyme *in vitro *(d)Result represented as mean ± S.D. of percent enzyme activity.**

The 50% ethanolic extract of *O. stamineus* showed a weak inhibition of the α-amylase enzyme. Figure 3 shows the percentage inhibition values of the 50% ethanolic extract of *O. stamineus*, sinensetin and acarbose against porcine α-amylase. The maximum inhibition was 69.2% at a concentration of 62.5 mg/ml (Figure 3(b)). Sinensetin produced a maximum inhibition of 85.8% at 2.5 mg/ml (Figure 3(c)). Acarbose showed a maximum percentage inhibition of 98.0% at 10 mg/ml (Figure 3(a)). The IC_50_ values for the 50% ethanolic extract of *O. stamineus*, acarbose and sinensetin were 36.70 ± 0.546 mg/ml, 4.89 ± 0.397 mg/ml and 1.13 ± 0.026 mg/ml, respectively (Figure 3(d)). IC_50_ values of sinensetin was significantly lower than acarbose and 50% ethanolic extract of *O. stamineus* at *P* < 0.05 and *P* < 0.001, respectively.

## Discussion

Diabetes mellitus is a metabolic disorder of multiple aetiologies that is characterized by chronic hyperglycaemia with disturbed carbohydrate, fat and protein metabolism resulting from defects in insulin secretion, insulin actions or both [22]. One therapeutic approach for treating diabetes is to decrease post-prandial hyperglycaemia. This is done by hindering the absorption of glucose through inhibition of the carbohydrate hydrolysing enzymes, α-amylase and α-glucosidase, in the digestive tract. Inhibitors of these enzymes delay carbohydrate digestion, causing a reduction in the rate of glucose absorption and consequently blunting the post-prandial increase of plasma glucose [23]. Examples of such inhibitors in clinical use are acarbose, miglitol and voglibose [24]. The post-prandial lowering of blood glucose by α-glucosidase inhibitors such as miglitol and acarbose after a starch load is well established. The mechanism of action is through the inhibition of the last step in carbohydrate digestion, namely the conversion of disaccharide to monosaccharide (glucose) and a consequent decrease in the rate of entry of glucose into the systemic circulation [25].

Present studies showed that *O. stamineus* extract significantly reduced plasma glucose concentration in both normal and diabetic rats [26,27]. In addition, *O. stamineus* extract also potentiated glucose-induced insulin secretion [28] may be beneficial to patients with diabetes mellitus who have a defect in the insulinotropic response to glucose. Although there are citations of antihyperglycaemic and antidiabetic [26,27] activities of *O. stamineus* extracts based on free radical scavenging activity and in part on increased glucose metabolism, there are no previous reports, at least to the best of our knowledge, about the inhibitory activity of this plant extract on in vitro α-glucosidase and α-amylase.

The present *in vitro* studies demonstrated an appreciable inhibitory activity of 50% ethanolic extracts of *O. stamineus* and sinensetin on α-glucosidase and α-amylase. *Orthosiphon stamineus* showed similar inhibitory effects on α-glucosidase and α-amylase. The IC_50_ values showed that the 50% ethanolic extract of *O. stamineus* and sinensetin show equal preference for both the α-glucosidase and α-amylase enzymes. Alpha-glucosidase and α-amylase inhibition could potentially be used as an effective therapy for post-prandial hyperglycaemia linked to type 2 diabetes. This approach has fewer side effects, such as abdominal distention, meteorism and possibly diarrhoea resulting from the abnormal bacterial fermentation of undigested carbohydrates in the colon, as normally seen when potent α-glucosidase inhibitors like acarbose and voglibiose are used. The goals of the *in vitro* α-glucosidase and α-amylase inhibitory studies were to provide *in vitro* evidence for the potential inhibition of α-glucosidase and α-amylase and to generate a stronger biochemical rationale for further studies on 50% ethanolic extracts of *O. stamineus* leaves.

Present study also found active anti-diabetic *O. stamineus* extract contain of high phenolic compound and flavonoid [27]. Several other classes of chemicals also have been found in *O. stamineus* that are rich terpenoids, caffeic acid derivatives and chromene [10,13,14]. Among these phytochemical groups caffeic acid and phenolic compound have been reported to increase glucose uptake in rat myocytes [28].

It is thought that one or more different compounds (e.g. sinensetin) that were present in the extracts play an important role in the inhibition of α-glucosidase and α-amylase. Furthermore, some of the compounds found in this extract (phenolics, flavonoids and their glycosides) were mentioned by Tadera *et al*[29] and Kwon *et al*[30] as being effective inhibitors of α-glucosidase. Based on the results presented in this study, it can be concluded that a 50% ethanolic extract of *O. stamineus* exerts an inhibitory effect on α-glucosidase and α-amylase. These results further support the traditional use of plants in medicine based on their inhibitory activity of glucose absorption in the gut.

It is thought that one or more different compounds (e.g. sinensetin) that were present in the extracts play an important role in the inhibition of α-glucosidase and α-amylase. Furthermore, some of the compounds found in this extract (phenolics, flavonoids and their glycosides) were mentioned by Tadera *et al* [29] and Kwon *et al* [30] as being effective inhibitors of α-glucosidase.

## Conclusion

Based on the results presented in this study, it can be concluded that a 50% ethanolic extract of *O. stamineus* exerts an inhibitory effect on α-glucosidase and α-amylase. These results further support the traditional use of plants in medicine based on their inhibitory activity of glucose absorption in the gut.

## Competing interests

The authors declare that they have no competing interests.

## Authors’ contributions

EAHM and MFY designed the study protocol, performed *in vitro* experiments and prepared the manuscript. MJAS and AS performed isolation of active compound. SHC, SCTand LFA performed LCMS/MS and HPLC studies. MZA designed the study protocol together with the first author. All authors read and approved the final manuscript.

## Pre-publication history

The pre-publication history for this paper can be accessed here:

http://www.biomedcentral.com/1472-6882/12/176/prepub
